# Multifunctionality of Polypyrrole Polyethyleneoxide Composites: Concurrent Sensing, Actuation and Energy Storage

**DOI:** 10.3390/polym12092060

**Published:** 2020-09-10

**Authors:** Nguyen Quang Khuyen, Rudolf Kiefer, Zane Zondaka, Gholamreza Anbarjafari, Anna-Liisa Peikolainen, Toribio F. Otero, Tarmo Tamm

**Affiliations:** 1 Faculty of Applied Sciences, Ton Duc Thang University, Ho Chi Minh City 700000, Vietnam; nguyenquangkhuyen@tdtu.edu.vn; 2 Intelligent Materials and Systems Lab, Institute of Technology, University of Tartu, Nooruse 1, 50411 Tartu, Estonia; zane.zondaka@ut.ee (Z.Z.); anna.liisa.peikolainen@ut.ee (A.-L.P.); tarmo.tamm@ut.ee (T.T.); 3 iCV Research Lab, Institute of Technology, University of Tartu, 50411 Tartu, Estonia; shb@ut.ee; 4 Faculty of Engineering, Hasan Kalyoncu University, 27410 Gaziantep, Turkey; 5 Centre for Electrochemistry and Intelligent Materials (CEMI), Universidad Politécnica de Cartagena, Aulario II, Paseo Alfonso XIII, E-30203 Cartagena, Murcia, Spain; toribio.fotero@upct.es

**Keywords:** polypyrrole materials, multifunctionality, sensing cations, electro-chemo-mechanical actuator, energy storage, actuating-sensing-supercapacitor device

## Abstract

In films of conducting polymers, the electrochemical reaction(s) drive the simultaneous variation of different material properties (reaction multifunctionality). Here, we present a parallel study of actuation-sensing-energy storage triple functionality of polypyrrole (PPy) blends with dodecylbenzenesulfonate (DBS-), PPy/DBS, without and with inclusion of polyethyleneoxide, PPy-PEO/DBS. The characterization of the response of both materials in aqueous solutions of four different salts indicated that all of the actuating, sensing and charge storage responses were, independent of the electrolyte, present for both materials, but stronger for the PPy-PEO/DBS films: 1.4× higher strains, 1.3× higher specific charge densities, 2.5× higher specific capacitances and increased ion-sensitivity towards the studied counterions. For both materials, the reaction energy, the material potential and the strain variations adapt to and sense the electrical and chemical (exchanged cation) conditions. The driving and the response of actuation, sensing and charge can be controlled/read, simultaneously, via just two connecting wires. Only the cooperative actuation of chemical macromolecular motors from functional cells has such chemical multifunctionality.

## 1. Introduction

Electro-chemo-mechanical actuators such as conducting polymers have been attracting wide interest for a wide range of applications [1] such as smart textiles [2], soft robotics [3], micro actuators [4], biomedical applications [5] and so on. One of the most studied among candidate materials has been films of the blends constituted by polypyrrole (PPy) and the macro-anion dodecylbenzenesulfonate (DBS^−^), PPy/DBS, electropolymerized from aqueous solutions. The macro-anions remain trapped in the film leading to exchange of cations, (C^+^), and solvent (S) for charge as well osmotic balance during oxidation/reduction of the film in liquid electrolytes, Equation (1) [6].

(1) $$[\left(PPy^{n+}\right)\left(DBS^{\;-\;}{)}_{n}\right]+\;n\left(C^{+}\right)+m\left(S\right)+\;n\left(e^{-}\right)\;\;\overset{\mathrm{red}}{\underset{\mathrm{ox}}{⇌}}\;[{\left(PPy\right)}^{0}{\left(DBS^{-}\right)}_{n}{\left(C^{+}\right)}_{n}\left(S{)}_{m}\right]$$

This reaction gives rise to cation-driven actuators: the material swells during reduction to solvated cation ingress (counterions) and contracts during oxidation of solvated counterions’ egress in direction of the electrolyte. Each PPy chain in the film acts as a multistep (n electrons extracted one by one) molecular machine and the cooperative conformational change of the individual polymer chains during a reaction gives rise to the material shape change (actuation). The events involved—electric pulse, exchange of ions and solvent, cooperative actuation of macromolecular chemical motors and material contraction/expansion—replicate those occurring in natural muscles. The energy of the reacting molecular motors adapts instantaneously to (senses) the ambient mechanical, chemical or thermal conditions. Various studies [7] of conducting polymers demonstrating concurrent actuation and sensing functions have been reported revealing that the motors can sense the electrolyte concentration [8], the working temperature [9], the mechanical loads on the actuator [10] and solvent of the electrolyte solution [11].

So far, the influence of the exchanged cations (ionic radius and solvation index) on both the actuation and sensing properties has received little attention. Here, we present a comparative electro-chemo-mechanical characterization of PPy/DBS and PPy-PEO/DBS films in the aqueous solutions of four different salts: LiCF_3_SO_3_, NaCl, EDMICF_3_SO_3_ and TMACl. The presence of a third component poly(ethylene oxide), (PEO) in the film has been shown to improve the ionic mobility [12] and the film conductivity [13]. It is expected that PEO will improve ion selectivity, sensing, actuation and energy storage functions simultaneously. The effect of PEO on the various properties of conducting polymers has been studied before, in terms of improved ion conductivity [13], increased strength, PEO blends of conducting polymers for the electrodes of Li batteries have shown increased charging/discharging properties and higher energy density [12].

To be sure that no irreversible side-processes take place during the oxidation/reduction cycles [14], the studied potential window was selected to be in the range of 0.65 to −0.6 V so that the charging/discharging processes commenced under charge balance (steady state) [15].

## 2. Materials and Methods

### 2.1. Materials

Sodium dodecylbenzenesulfonate (NaDBS, 99%), poly (ethylene oxide) (PEO, Average Mw ca. 100,000 g/mol), ethylene glycol (EG, 99.8%), deionized water (Milli-Q), lithium trifluoromethanesulfonate (LiCF_3_SO_3_, >99.9%), 1-ethyl-2,3-dimethylimidazolium trifluoromethanesulfonate (EDMICF_3_SO_3_, 95%), tetramethylammonium chloride (TMACl, 95%) and sodium chloride (NaCl, technical grade) were acquired from Sigma-Aldrich (Taufkirchen, Germany) and used without further purifications. Pyrrole (Py ≥98%) from Sigma-Aldrich (Taufkirchen, Germany) was purified and stored in a fridge under nitrogen atmosphere.

### 2.2. Electrochemical Synthesis of PPy Blends

The PPy-PEO/DBS (5 wt% PEO in polymerization solution) and PPy/DBS films were electrogenerated by galvanostatic polymerization (constant current density of 0.1 mA cm^−2^ at −20 °C for 40,000 s (11.1 h)), controlled by a potentiostat (PARSTAT 2273, Princeton applied research, Oak Ridge, TN, USA) in a two-electrode electrochemical cell on stainless steel working electrodes (surface area 18 cm^2^) with a platinum mesh (2.5 × 2.5 cm) counter electrode. The cell contained the polymerization solution of: 0.1 M Py and 0.1 M NaDBS, or 0.1 M Py and 0.1 M NaDBS and 5%, *w*/*w* PEO in an EG:Milli-Q (ethylene glycol: deionized water) (1:1) mixture. Each electrochemically deposited film was removed from stainless steel electrode, washed several times in ethanol and Milli-Q to remove any residual pyrrole and NaDBS. The films were placed in an oven (40 °C, 24 h, 2 mbar) and stored in dry state for further use, the thickness of PPy/DBS and PPy-PEO/DBS films was 28 ± 2 µm and 32 ± 2 µm, respectively, measured using an Electronic Micrometre Gauge Meter (Dainu, 0.001 mm sensitivity).

### 2.3. Linear Actuation

At least three batches of each PPy material was polymerized, and samples prepared and characterized under each studied experimental condition, showing the results in mean values with standard deviations.

**Scheme 1:**
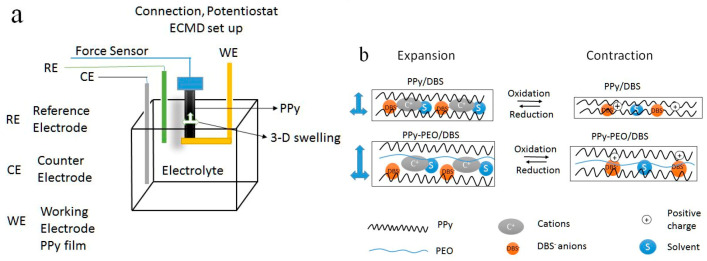
(**a**): Electrochemical cell with the working electrode (WE) the polypyrrole (PPy) films located between the force sensor clamps, the Pt counter-electrode (CE) and the reference electrode Ag/AgCl (3 M KCl) (RE). (**b**): Mechanism of oxidation/reduction triggered contraction/expansion with legends as block chains PPy (black) and poly(ethylene oxide) (PEO) chains (blue) involving immobile anions “DBS^−^“ (dodecylbenzenesulfonate), cations “C^+^”, solvent “S” and positive charges “+”.

Each electrogenerated film was cut into strips (1.2 × 0.1 cm) and anchored between a force sensor (TRI202PAD, Panlab, Barcelona, Spain) and a lower arm provided with gold wires serving as the working electrode in a three-electrode cell (Scheme 1a) with schematic mechanism of volume change during oxidation/reduction of PPy films (Scheme 1b). The counter electrode was a platinum sheet (2.5 × 2 cm), the reference electrode an Ag/AgCl (3 M KCl). The cell contained a 0.2 M aqueous solution of one of the studied salts: LiCF_3_SO_3_, EDMICF_3_SO_3_, TMACl or NaCl. The films were submerged in the electrolyte with pre-stretching of 1% (equivalent to 200 mg) for 6 h before the measurements. The electro-chemo-mechanical response of the films was studied in potential range of 0.65 to −0.6 V by consecutive potential cycles (cyclic voltammetry, scan rate 5 mV s^−1^), consecutive square wave potential steps, or consecutive square wave current steps using a potentiostat Biologic PG581 (Göttingen, Germany). Independent of the used methodology, the reversible oxidation/reduction of the material promoted a reversible length change (under a constant applied force of 9.8 mN) was conducted with the in-house software of the ECMD (electro-chemo-mechanical deformation) device [16]. The strain, ε in %, was obtained from the formula ε = ΔL/L*100%, where ΔL = L − L_1_ was the length change, with L the original length of the film, and L_1_ the length at any time point during the deformation. The energy storage properties of the films were studied by chronopotentiometry.

### 2.4. Characterization

Scanning electron microscopy (Helios NanoLab 600, FEI, Oregon, OR, USA) was performed of the PPy/DBS and PPy-PEO/DBS samples. To appraise the ion content, energy-dispersive X-ray (EDX) spectroscopy (Oxford Instruments with X-Max 50 mm^2^ detector, High Wycombe, UK) of the samples was performed in oxidized (polarized at 0.65 V for 5 min) and reduced (polarized at −0.6 V for 5 min) state. The resistivity “R” of the films was measured with the two-point probe with a surface resistivity meter (Guardian SRM, Oxbow Circle, Cocoa, FL, USA) at different locations over a 0.1 cm distance. The electronic conductivity σ (Equation (2)) was calculated using the cross-section area “A” with length “l” of the samples.

(2) $$\sigma =\;\frac{l}{\left(R*A\right)}$$

## 3. Results and Discussion

As mentioned above, PEO has been shown to be able to improve the ionic mobility [12] and with it, the film conductivity [13] of conducting polymers. The main goal of the present research was to investigate whether PEO can also be beneficial to the whole set of concurrently appearing features of electro-chemo-mechanically responsive conducting polymer films, like ion selectivity, sensing, actuation and energy storage functions.

**Figure 1:**
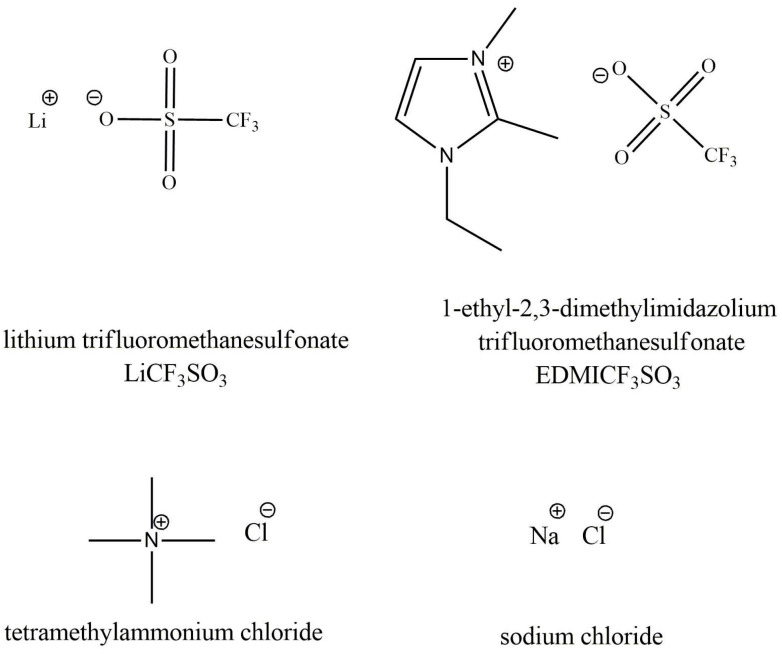
Structures of the ions of the salts considered as the electrolytes.

The PPy/DBS and PPy-PEO/DBS linear films were mainly cation driven, following reaction 1. The immobile DBS^−^ anions inside the PPy film led during reduction to solvated cation entrance and solvent induced over osmotic balance, bringing along the concomitant reaction-driven material expansion. The PEO added during the electropolymerization process increases the ionic conductivity in the PPy-PEO/DBS in contrast to the PPy/DBS films [13]. As long as the structural features are not compromised by the addition, one should expect improved linear actuation—larger strain and faster response. It has not been determined if the improvement be influenced on the nature of the electrolyte ions. Figure 1 shows the structure of the different ions considered here to check the electro-chemo-mechanical responses from the materials in different electrolytes.

In aqueous solution the cations carry different hydration numbers. The small and high-charge-density Li^+^ ion (ion radius 0.185 nm) has a hydration number of 4 [17], while Na^+^ ions (van der Waals radius 0.227 nm) are surrounded by 5 water molecules [17]. The hydration shell of organic cations such as EDMI^+^ (van der Waals radius 0.352 nm) is supposed to be weak due to the hydrophobic methyl and ethyl ligands [18], restricting solvent access to the charge. The TMA^+^ ion (van der Waals radius 0.322 nm) also has high hydrophobicity [19,20] and poor charge accessibility, being exchanged as a single entity between the PPy network and the electrolyte. During the reaction-driven exchange of cations (Reaction 1) the solvent still moves with the cations to keep the osmotic balance.

### 3.1. Characterization

#### 3.1.1. Morphology and Conductivity of Polypyrrole/Dodecylbenzenesulfonate (PPy/DBS) and PPy-PEO (Poly(ethylene Oxide))/DBS Operated in Different Electrolytes

**Figure 2:**
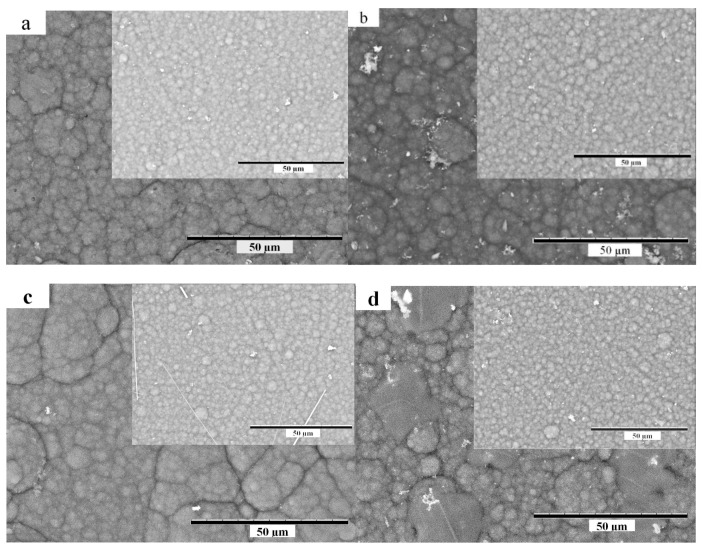
Scanning electron microscopy (SEM) surface images (scale bar 50µm) of PPy-PEO/DBS (inset PPy/DBS) after actuation cycles (stored in oxidized state of 0.65 V) in aqueous electrolyte (**a**): LiCF_3_SO_3_, (**b**): NaCl, (**c**): EDMICF_3_SO_3_ and (**d**): TMACl.

SEM surface images of PPy/DBS and PPy-PEO/DBS operated in different electrolytes are presented in Figure 2.

There were no distinct differences of the morphologies of the materials caused by the different electrolytes (Figure 2). However, the effect of the addition of PEO can be clearly observed, as the morphology of PPy-PEO/DBS is significantly rougher than that of PPy/DBS, as expected [21]. The conductivity of the PPy/DBS and PPy-PEO/DBS films (in oxidized state) was determined (Equation (2)) straight after polymerization and then after actuation (150 actuation cycles) in different electrolytes, the results are shown in Table 1.

Table 1. shows that the conductivity of the oxidized dry materials is high after polymerization dropping to about one half after actuations in different electrolytes due to the lower potential reached, as seen before [22,23]. In all cases, the conductivity of PPy-PEO/DBS films was about double that of the corresponding PPy/DBS films, demonstrating that PEO has a significant positive effect on the conductivity independent of the electrolyte. Higher conductivity should enable faster charging/discharging reactions during the redox cycles; therefore, we could expect the incorporation of more counter-ions (in the same time frame) leading to improved actuation response.

#### 3.1.2. Energy-Dispersive X-ray (EDX) Spectroscopy

**Figure 3:**
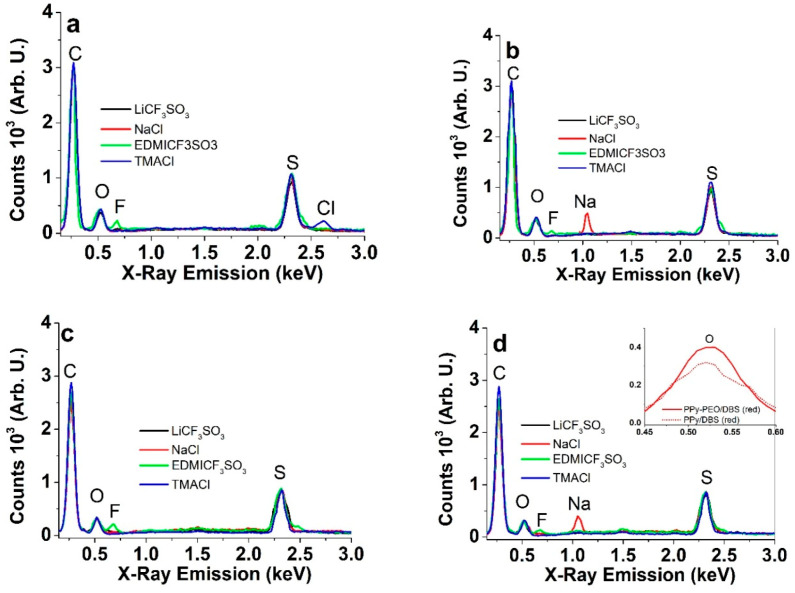
Energy-dispersive X-ray (EDX) spectra of PPy films in the oxidized (polarized 5 min at 0.65 V) and reduced state (5 min −0.65 V) in aqueous electrolytes of LiCF_3_SO_3_ (black), NaCl (red), EDMICF_3_SO_3_ (green) and TMACl (blue): (**a**): oxidized PPy-PEO/DBS, (**b**): reduced PPy-PEO/DBS; (**c**): oxidized PPy/DBS; and (**d**): reduced PPy/DBS. The inset in (d) compares the counts of the oxygen peak of PPy-PEO/DBS (red, solid line) to PPy/DBS (red, dotted) at reduced state, operated in aqueous NaCl electrolyte.

To evaluate the ion content, and its variation in PPy-PEO/DBS and PPy/DBS, the EDX spectroscopy was performed on films oxidized and reduced in different electrolyte solutions (Figure 3).

**Figure 4:**
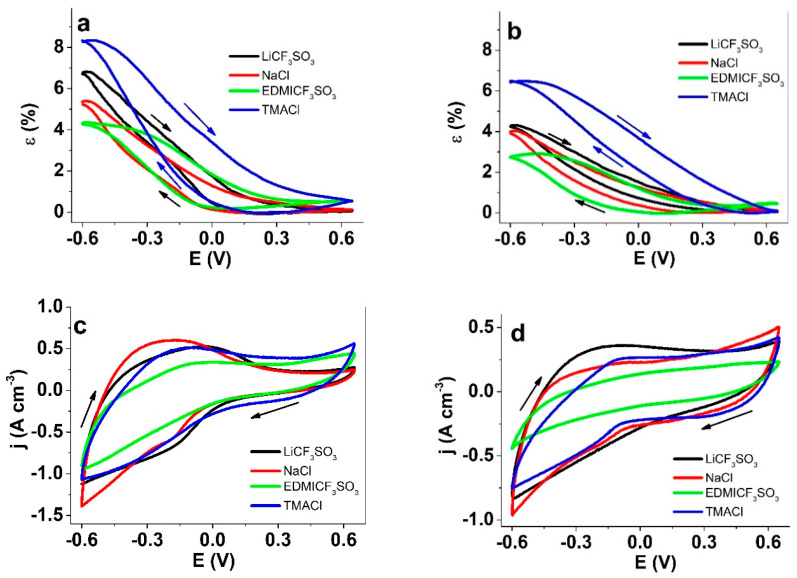
Steady state responses of films submitted to potential cycles (scan rate 5 mV s^−1^) between 0.65 to −0.6 V against Ag/AgCl (3 M KCl) in aqueous solutions of LiCF_3_SO_3_ (black), NaCl (red), EDMICF_3_SO_3_ (green) and TMACl (blue) (**a**): strain, ε, of PPy-PEO/DBS films; (**b**): strain, of PPy/DBS; (**c**): current density, j, of PPy-PEO/DBS films; and (**d**): current densities, of PPy/DBS films. The arrows indicate the scan direction.

In Figure 3a–d, the typical signals for carbon (C) at 0.27 keV, oxygen (O) at 0.52 keV, fluorine (F) at 0.68 keV, sodium (Na) at 1.05 keV, sulfur (S) at 2.32 keV and chlorine (Cl) at 2.62 keV can be seen. The intensity due to the organic cations cannot really be assigned. The spectra of oxidized (Figure 3a) and reduced PPy-PEO/DBS (Figure 3b) in LiCF_3_SO_3_ appear identical, as Li was not detectable in EDX. Consequently, only Li^+^ ions moved in and out of the films during the redox cycles. In the case of EDMICF_3_SO_3_, a fluorine peak increased upon oxidation and decreased upon reduction, which means that the anions CF_3_SO_3_^−^ participated in charge balancing with some of them even becoming immobile and staying in the PPy network upon reduction. In the case of NaCl as the electrolyte, a strong Na peak was observed upon reduction, disappearing upon oxidation. Somewhat unexpectedly, for PPy-PEO/DBS in TMACl electrolyte, a chlorine peak appeared upon oxidation, which was not observed for PPy/DBS. Otherwise the trends of PPy/DBS showed a similar tendency of anion and cation involvement upon oxidation and reduction. The inset of Figure 4d shows the increase of O peak for PPy-PEO/DBS compared to PPy/DBS (same for all other electrolytes as well), corresponding to the incorporated PEO [21].

### 3.2. Linear Actuation Controlled by Cyclic Voltammetry

The strain variation of PPy-PEO/DBS and PPy/DBS films submitted to cyclic voltammetry between 0.65 and −0.6 V at the scan rate of 5 mV s^−1^ in different aqueous electrolyte solutions is shown in Figure 4a,b, respectively. Figure 4c,d present the voltammetric responses, as evolutions of the current density of PPy-PEO/DBS and PPy/DBS films, respectively. The coulovoltammetric results (Figure S1a,b) show the evolution of charge density from PPy-PEO/DBS and PPy/DBS films.

Qualitatively, the strain response of both materials was rather similar with rising strains (swelling) upon reduction and contraction (decreasing strain) upon oxidation. The reaction-induced reversible strain variation of PPy-PEO/DBS films was 1.3 times higher than that of the PPy/DBS films, whatever the studied electrolyte. The strain difference was directly related to the involved charges (Figure S1) which drive (Reaction 1) the exchanged counterions into the film, bringing along a volume variation. This higher reactivity can be related to the higher ionic conductivity of PPy-PEO/DBS films [13] (Table 1). The decreasing order of the studied electrolytes by strain variation in PPy-PEO/DBS was: TMACl (8.4%) > LiCF_3_SO_3_ (6.8%) > NaCl (5.2%) > EDMICF_3_SO_3_ (4.3%).

For PPy/DBS films, the order was qualitatively the same, with TMACl (6.4%) > LiCF_3_SO_3_ ≈ NaCl (4.1%) > EDMICF_3_SO_3_ (2.7%).

It is interesting to note that PPy-PEO/DBS films (Figure 4a) in TMACl and EDMICF_3_SO_3_ solutions showing slight mixed actuation with a small expansion upon oxidation in the range of 0.5–0.6%. The existence of Cl^−^ and a fluoride peak (F) in EDX at oxidation at Figure 3a confirms that the anions were incorporated. While there can be several possible explanations to the aforementioned difference in behavior, it is obvious that the distinction runs along the hydrophobic-hydrophilic line of cations. Apparently, the more hydrophobic (and with sterically more hidden charge centers) cations like TMA^+^ and EDMI^+^ are less effectively transported through the PEO-embedded PPy matrix, as perhaps most clearly attested by the less steep onsets of the oxidation peaks (Figure 4c). Hence, the flux of cations is not sufficient to maintain charge neutrality, and a small part of the net charge on the PPy chains is compensated for by the anions. For PPy/DBS, the situation is slightly different, as the actuation TMACl is purely cation-driven, a small expansion upon oxidation is only observed in EDMICF_3_SO_3_– containing the bulkier of the two organic cations EDMI^+^. Although the current density plots (Figure 4d) suggest there might be anion participation, the possible anion flux does not bring along enough volume change in the matrix to cause observable macroscopic dimensional change.

As seen from the voltammetric responses in Figure 4c,d, the PPy-PEO/DBS films demonstrated the 1.2 times higher current densities (higher film oxidation/reduction charges), whatever the studied electrolyte. Among the studied salts, the EDMICF_3_SO_3_ solution gives the lowest current densities. There are also qualitative differences, as in case of PPy-PEO/DBS, the oxidation sets in earlier, at already −0.15 V, compared to −0.04 V for the other salts. However, for PPy/DBS the trends are not as clearly distinguishable. While the charge density (Figure S1a,b) in EDMICF_3_SO_3_ was the lowest of all salts for both PEO-containing (75 vs. 97 C cm^−3^) and not containing films (40 vs. 74 C cm^−3^), and the difference is much clearer in the plots for PPy/DBS, which may be related to the relatively poorer conductivity. The attained closed loops of the charge density plots inside the studied potential range confirmed both the reversibility of the oxidation/reduction charges and the so called “steady state” which means that we obtain the same coulovoltammetric response from consecutive potential cycles in every electrolyte [15].

### 3.3. Energy Storage

**Figure 5:**
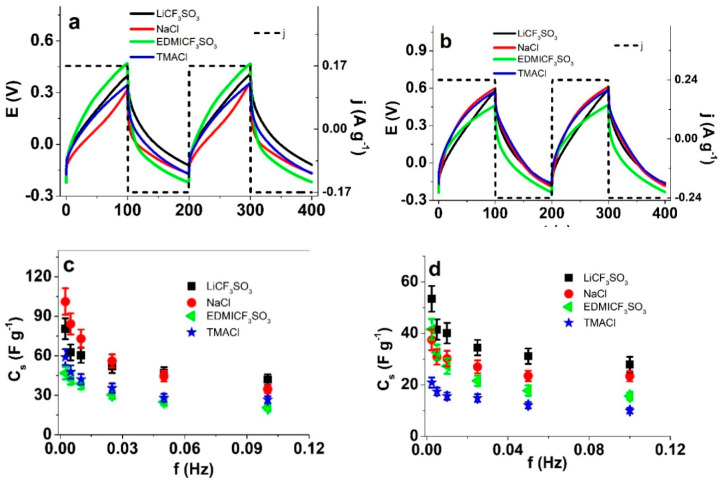
Chronopotentiometric responses to square current density waves for PPy-PEO/DBS (±0.085 A g^−1^, ±0.17 A g^−1^, ±0.34 A g^−1^, ±0.85 A g^−1^, ±1.7 A g^−1^ and ±3.4 A g^−1^) and PPy/DBS (±0.12 A g^−1^, ±0.24 A g^−1^, ±0.48 A g^−1^, ±1.2 A g^−1^, ±2.4 A g^−1^ and ±4.8 A g^−1^) at frequencies 0.0025 Hz, 0.005 Hz, 0.01 Hz, 0.025 Hz, 0.05 Hz and 0.1 Hz in different aqueous electrolytes (LiCF_3_SO_3_ (black line), NaCl (red line), EDMICF_3_SO_3_ (green line) and TMACl (blue line)) showing the potential time curves with current density j of (**a**): PPy-PEO/DBS (frequency 0.005 Hz, current density ±0.17 Ag^−1^) and (**b**): PPy/DBS films (frequency 0.005 Hz, current density ±0.24 Ag^−1^). The specific capacitance C_s_ obtained from Equation (3) against the frequency in LiCF_3_SO_3_ (■), NaCl (●), EDMICF_3_SO_3_ (◄) and TMACl (★) are shown in (**c**): for PPy-PEO/DBS and (**d**): PPy/DBS films.

Composites of conducting polymers (with and without inorganic salts [23] or carbon materials [24]) have been investigated as possible electrodes for supercapacitors. Figure 5a,b show the steady state chronopotentiometric (charge/discharge) responses to consecutive square current waves (±0.17 A g^−1^ for PPy-PEO/DBS; ±0.24 A g^−1^ for PPy/DBS) at 0.005 Hz in each of the studied electrolytes. The experiments were repeated at different frequencies. Taking into account the ohmic drop, the slope and the applied current density “j” (A g^−1^) allow following Equation (3) [25], to determine the specific capacitance C_s_ of the PPy materials.

(3) $$C_{s}=\;\frac{j}{-slope}$$

Figure 5c,d present the results attained at different frequencies from the two studied PPy materials.

The attained specific capacitances, C_s_, from the studied electrolytes were higher, whatever the applied frequency, using PPy-PEO/DBS films (Figure 5c) than using PPy/DBS films (Figure 5d). The PPy-PEO/DBS film presents the highest specific capacitance at 0.0025 Hz (101.2 ± 9.8 F g^−1^ in NaCl solution) followed by 80.5 ± 7.8 F g^−1^ in LiCF_3_SO_3_ solutions. Similar values, 99 F g^−1^ have been obtained for a PPy-CNT/DBS composite in aqueous solutions [26]. For the same best two electrolytes (NaCl, LiCF_3_SO_3_), the attained specific capacitances using PPy/DBS films at the same 0.0025 Hz frequency, were 38.7 ± 3.9 F g^−1^ and 53.4 ± 5.5 F g^−1^, respectively. It has been suggested that the generation of PPy films by pulse technique can increase the specific capacitance in acid solutions [27] up to 400 F g^−1^. Without specific optimization, the PPy-PEO/DBS films still present very reasonable steady state (under cycling) specific capacitance values.

### 3.4. Sensor Calibration

Intense studies have been carried out in the past [9,11,28] to verify concurrent actuation and sensing properties of chemically [10] and electrochemically [29] polymerized PPy films. The evolution of the energy devoured by the reaction driving the actuator adapts to (senses) the energetic conditions (electrical, thermal, mechanical or chemical). The conducting polymer reactions, therefore, drive simultaneously several sensors and an actuator (dimensional changes). The most paradigmatic situation related to modern technologies is that both actuating signals (flowing current and charge determining the movement rate and linear displacement) and sensing signals (reaction energy or any of its components, potential and charge) are simultaneously present in the only two connecting wires. The ensemble replicates the natural muscles’ actuation: the brain is also connected to each muscle by two neurons, the motor neuron drives the actuation orders from the brain and the sensory neuron sends the information about the mechanical, chemical and thermal working conditions from the muscle back to the brain. In both natural and artificial systems, sensing never interferes with the actuation because it is a consequence of the reaction-driven actuation. Here the PPy-PEO/DBS films were contradicted to consecutive square waves of current density keeping the same anodic/cathodic (oxidation/reduction) charge 17 C g^−1^ (±0.085 A g^−1^, ±0.17 A g^−1^, ±0.34 A g^−1^, ±0.85 A g^−1^, ±1.7 A g^−1^ and ±3.4 A g^−1^) at frequencies (0.0025 Hz, 0.005 Hz, 0.01 Hz, 0.025 Hz, 0.05 Hz and 0.1 Hz), respectively. The procedure was repeated in the four studied electrolytes. Our aim was to check if the energy consumed during the attained stationary chronopotentiometric responses (i.e., Figure 5a) can provide information about (can sense) either, the applied specific current, the applied frequency, of the cation exchanged during the reaction.

The electric energy consumed by the reaction (polymer oxidation or reduction) at any time of the current flow, U_e_ (t), is given by Equation (4).

(4) $$U_{e}\left(t\right)=\;j\int E\left(t\right)dt$$

The potential evolution during the cathodic current flow [E(t) during discharging] was integrated and multiplied by the specific current, j (applied current, I, divided by the polymer mass, A g^−1^) to obtain the specific electrical energy (U_e_, Jg^−1^) consumed during the reaction.

**Figure 6:**
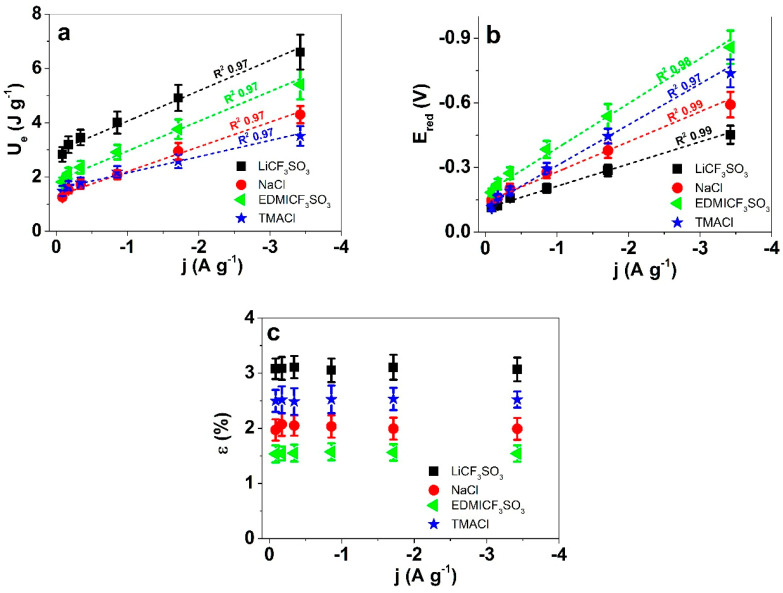
Chronoamperometry of PPy-PEO/DBS linear films in various aqueous electrolytes LiCF_3_SO_3_ (■), NaCl (●), EDMICF_3_SO_3_ (◄) and TMACl (★) showing in (**a**): the electrical Energy U_e_, in (**b**): the potential E_red_ at reduction and in (**c**): the linear strain ε against the current density j (A g^−1^) at reduction. The dashed lines in (**a**) and (**b**) represents the linear fit with included correlation coefficient R^2^.

Figure 6a shows virtually linear evolution (correlation coefficients, R^2^ = 0.97) of the consumed specific electrical energy during the oxidation/reduction of PPy-PEO/DBS films in relation to the applied current density in each of the studied electrolytes. Therefore, in every electrolyte the specific energy consumed during actuation is a good sensor of the flowing current, and the concomitant line, and the respective equation, can be used as the calibration line and the sensing equation, respectively. Interestingly, the sensitivity depends on the electrolyte, as TMACl solutions present the lowest sensitivity (slope). The other three electrolytes share a similar sensitivity and at a particular concentration, one can identify the nature of the exchanged cations by the consumed specific reaction energy.

Figure 6b collects the evolutions of the material potential at the end of the reduction (minima in Figure 6a) chronopotentiometric responses as a function of the applied specific currents. The attained linear relationships (correlation coefficients greater than 0.97) indicate that the material potential is an excellent sensor of the flowing current. The slopes (J.A^−1^): −0.1031 ± 0.005 from LiCF_3_SO_3_ solutions; 0.14 ± 0.008 from NaCl; 0.19 ± 0.013 from TMACl and 0.208 ± 0.014 from EDMICF_3_SO_3_ solutions are different for the studied electrolytes (LiCF_3_SO_3_ < NaCl < TMACl < EDMICF_3_SO_3_). Those facts indicate that: as an electrical sensor the material reaction presents the higher sensibility in EDMICF_3_SO_3_ aqueous solutions and, as a chemical sensor the material reaction allows the identification of the cation involved in the reaction.

Figure 6c depicts the strain variation (actuation response) of PPy-PEO/DBS in the four studied electrolytes. As expected, consuming the same oxidation/reduction charge the strain variation is a constant, and different for each studied electrolyte following the sequence: LiCF_3_SO_3_ > TMACl > NaCl > EDMICF_3_SO_3_. For the same reaction charge, the number of counterions exchanged to balance the polymeric charges are the same, whatever the electrolyte used, but the diameter of the exchanged ions is different and the number of water molecules exchanged for maintaining osmotic balance is also different. As a result, for the same specific charge driving the reaction, the strain variation in each electrolyte is different. The strain variation for the same applied specific current can also allow the identification of the used electrolyte.

Similar sensing figures were attained using PPy/DBS films, showing the results for the consumed specific electrical energy in Figure S2a, for the potential E_red_ in Figure S2b and for the actuation response in Figure S2c. However, the separation was much weaker in PPy/DBS, indicating that the “wires”—the conducting polymer matrix—should have as high conductivity as possible, in order to allow the sensor functions to clearly establish. As seen previously [21], the diffusion coefficients upon reduction in PPy-PEO/DBS can be nearly double of those of PPy/DBS. A faster exchange of ions allows the equilibria to be established faster, leading to more clear and controlled dependencies. The filtering effect of PEO supporting cation transport and suppressing anion transport likely also plays a role here.

Both results corroborate that being faradaic actuators, in conducting polymers the charge density determines the actuation responses [8]. The chronoamperometric responses at different current densities and frequencies, for the same charge (here 17 C g^−1^) give, therefore, a constant actuation response [30] in each of the studied electrolytes. The described actuating sensors can detect different (cat)ions in aqueous solutions, becoming a useful tool for water quality control. This is of remarkable interest regarding organic cations such as TMA^+^ or EDMI^+^, which have certain toxicity for marine life [31,32] but have been rather difficult to detect. Figure 6a reveals that the electrical energy U_e_ from Equation (4) shows a clear separation between the studied electrolytes (different cations). Table 2 represents the parameters of the linear equations for the consumed electrical energy and potential evolution at the end of the reduction, as well as the strain in each applied electrolyte.

Comparing the material potential at the end of the reduction processes, a good separation with different slopes (good sensibilities) can be observed using PPy-PEO/DBS films in different electrolytes (Figure 6b, Table 2), and much better than using PPy/DBS films (Figure S2b). Therefore, the improved conductivity and other related properties of PPy-PEO/DBS also provide a clear benefit for potential sensor applications in terms of sensing organic and metal ions in aqueous solutions.

Figure 6c shows the evolution of the strain attained during the cathodic processes (film reduction) versus the applied current density, at a constant charge consumption of 17 C g^−1^. Being constant, the charge also determines the strain response to be the same in each of the studied electrolytes (same number of exchanged counterions between the film and the electrolyte). The strain, however, varies between the electrolytes (different radios of the exchanged ions and different number of solvent molecules exchanged per unit of charge) in the order: LiCF_3_SO_3_ > TMACl > NaCl > EDMICF_3_SO_3_. By comparing those results with Figure S2c, it is obvious that PPy/DBS has lower sensing abilities than PPy-PEO/DBS.

A potential application for the multifunctional PPy-PEO/DBS films can be found in sweat analyzers/sensors [33] detecting sodium levels over change in potentials or strain, whereas the moderate energy storage can be applied for battery charging to send signals in case of the critical levels to a mobile device or straight to a nearby doctor. The sensitivity of the sensor materials introduced here can be improved by focusing on the particular function of the material. The interplay with other factors influencing the sensitivity, like pH, temperature and solution composition of the sensing ability of PPy-PEO/DBS films will be studied in a future work.

## 4. Conclusions

An empirical study was presented showing the multifunctional (actuating/sensing/charge storage) properties of PPy-PEO/DBS films, which were compared with classical pristine PPy/DBS films. The added PEO component improved both conductivity and ionic mobility, allowing the material to reach higher oxidation/reduction charges than those available to PPy/DBS films, whatever the studied electrolyte. As a consequence, for the same voltammetric stimulus, higher (1.4 times) strain variations were reached by PPy-PEO/DBS films. In addition, related to the energy storage, the specific capacitances reached around 2.5 times higher in PPy-PEO/DBS films (100 F g^−1^ in NaCl aqueous solutions). While both materials demonstrated concurrent sensing and actuating properties responding to the presence of different electrolytes under the flow of different specific currents, the PPy-PEO/DBS films showed higher chemical and electrical sensitivities. Good sensing calibration lines were attained for the response of the reaction energy and material potential related to the applied current, changing both energy and potential values and/or slopes as a function of the studied electrolytes.

Overall, both PPy-PEO/DBS and PPy/DBS films behave in each of the studied electrolytes simultaneously as an actuator (strain change), a supercapacitor (specific capacitance evolution), an electrical sensor (flowing current), and a chemical sensor (involved cation). The actuation signals (current and charge), storage signals (charge) and sensing signals (reaction energy, material potential) are present at any reaction moment in only the two connecting wires. One reaction drives, simultaneously, four functions in one material, replicating the biological functions based on the cooperative actuation of chemical or electrochemical molecular machines.

## Figures and Tables

**Table 1:** Conductivity of PPy-PEO/DBS and PPy/DBS after polymerization (oxidized state) and after actuation (oxidized state) in aqueous electrolytes of LiCF_3_SO_3_, NaCl, EDMICF_3_SO_3_ and TMACl.

Electrolyte	PPy-PEO/DBS (S cm^−1^)	PPy/DBS (S cm^−1^)
Direct after Polymerization	17.7 ± 1.3	7.9 ± 0.6
LiCF_3_SO_3_	9.3 ± 0.4	4.8 ± 0.2
NaCl	8.6 ± 0.6	4.6 ± 0.3
EDMICF_3_SO_3_	6.3 ± 0.3	2.8 ± 0.2
TMACl	9.0 ± 0.5	4.4 ± 0.3

**Table 2:** Linear sensing equations of the flowing current from: the consumed electrical energy U_e_, the material potential after reduction, E_red,_ and the maximum strain variation, ε, using PPy-PEO/DBS films in solutions of LiCF_3_SO_3_, NaCl, TMACl, and EDMICF_3_SO_3_ under the conditions described in Figure 5.

Electrolytes	Linear Equation of Electrical Energy U_e_ (J g^−1^) =	Linear Equation of Potential at Reduction −E_red_ (V) =	Strain ε [%]s
LiCF_3_SO_3_	−1.12 ± 0.09 j (A g^−1^)	0.1031 ± 0.005 j (A g^−1^)	3.1 ± 0.25 j
NaCl	−0.91 ± 0.07 j (A g^−1^)	0.14 ± 0.008 j (A g^−1^)	2.02 ± 0.14 j
TMACl	−0.60 ± 0.04 j (A g^−1^)	0.19 ± 0.013 j (A g^−1^)	2.51 ± 0.17 j
EDMICF_3_SO_3_	−1.09 ± 0.08 j (A g^−1^)	0.208 ± 0.014 j (A g^−1^)	1.55 ± 0.09 j

## Supplementary Materials

The following are available online at https://www.mdpi.com/2073-4360/12/9/2060/s1, Figure S1: Cyclic voltammetry (scan rate 5 mV s^−1^) in potential range 0.65 to −0.6 V against Ag/AgCl (3 M KCl) of PPy films operating in aqueous electrolytes of LiCF_3_SO_3_ (black line), NaCl (red line), EDMICF_3_SO_3_ (green line) and TMACl (blue line) showing the charge density Q against potential E of a: PPy-PEO/DBS and b: PPy/DBS films, Figure S2: Chronoamperometry of PPy/DBS linear films at varied current densities (±0.12 A g^−1^, ±0.24 A g^−1^, ±0.48 A g^−1^, ±1.2 A g^−1^, ±2.4 A g^−1^ and ±4.8 A g^−1^) and frequencies (0.0025 Hz, 0.005 Hz, 0.01 Hz, 0.025 Hz, 0.05 Hz and 0.1 Hz) in different aqueous electrolytes LiCF_3_SO_3_ (■), NaCl (●), EDMICF_3_SO_3_ (◄) and TMACl (★) showing in **a**: the electrical Energy U_e_, in **b**: the potential E_red_ at reduction and in **c**: the linear strain ε against the current density j at reduction.
