# Supplementary material for: Multifunctionality of Polypyrrole Polyethyleneoxide Composites: Concurrent Sensing, Actuation and Energy Storage
Source: Polymers (Basel). 2020 Sep 10;12(9):2060. doi: 10.3390/polym12092060 (PMC7576489; doi:10.3390/polym12092060)
Supplement: Supplementary file 1 [file polymers-12-02060-s001.pdf]

polymers

Supplementary:

Multifunctionality of Polypyrrole Polyethyleneoxide Composites: Concurrent Sensing, Actuation and Energy Storage

Nguyen Quang Khuyen [1], Rudolf Kiefer [1,*], Zane Zondaka [2], Gholamreza Anbarjafari [3,4], Anna-Liisa Peikolainen [2], Toribio F. Otero [5] and Tarmo Tamm [2]

Received: 22 August 2020; Accepted: 8 September 2020; Published: date

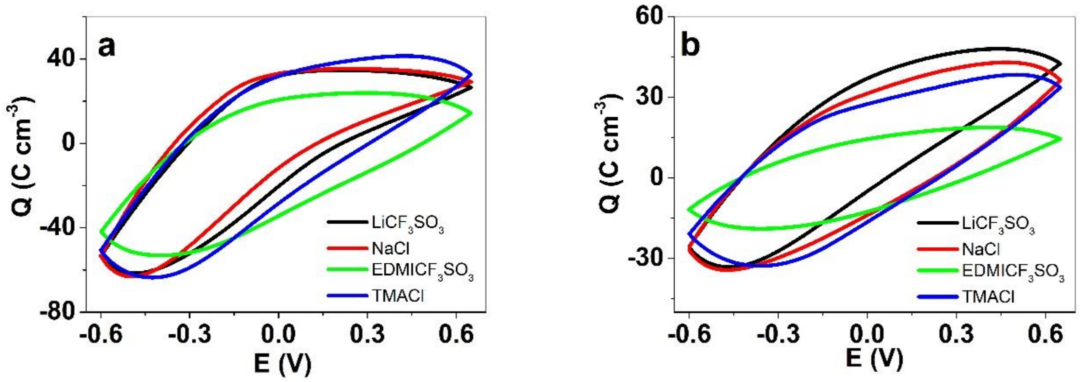

Figure S1. Cyclic voltammetry (scan rate 5 mV s^{-1}) in potential range 0.65 to −0.6 V against Ag/AgCl (3 M KCl) of PPy films operating in aqueous electrolytes of $LiCF_3SO_3$ (black line), NaCl (red line), $EDMICF_3SO_3$ (green line) and TMACl (blue line) showing the charge density Q against potential E of **a**: PPy-PEO/DBS and **b**: PPy/DBS films.

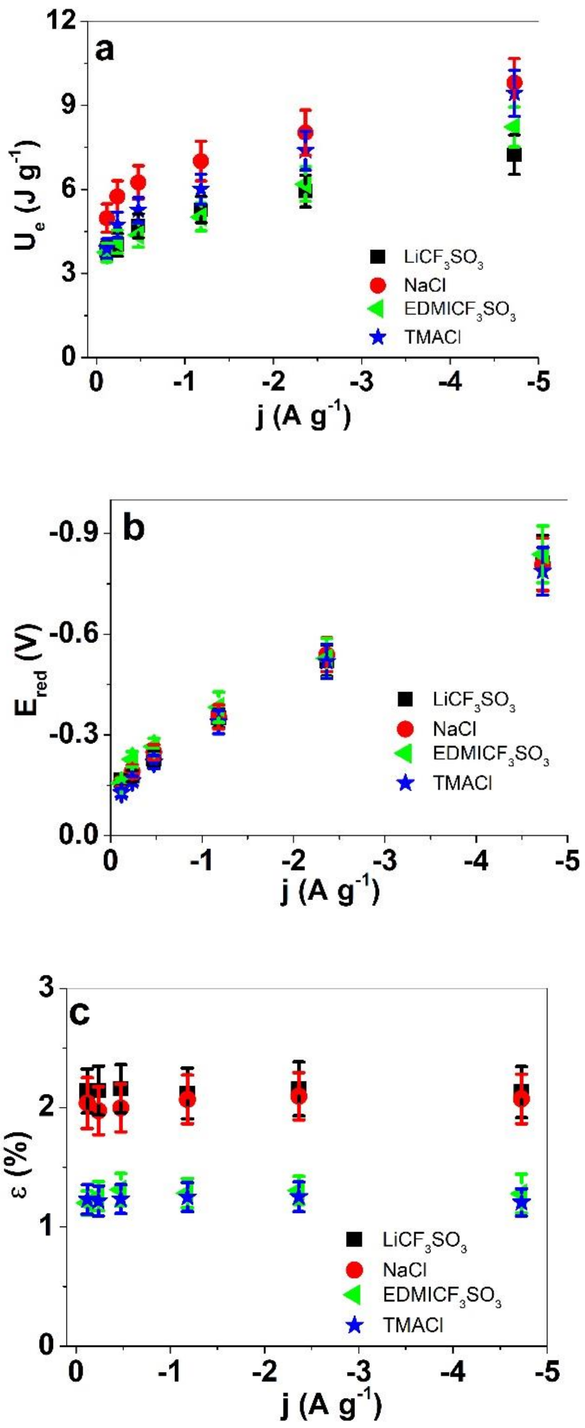

Figure S2. Chronoamperometry of PPy /DBS linear films at varied current densities (±0.12 A g^{-1}, ±0.24 A g^{-1}, ±0.48 A g^{-1}, ±1.2 A g^{-1}, ±2.4 A g^{-1} and ±4.8 A g^{-1}) and frequencies (0.0025 Hz, 0.005 Hz, 0.01 Hz, 0.025 Hz, 0.05 Hz and 0.1 Hz) in different aqueous electrolytes $LiCF_3SO_3$ (■), NaCl (●), $EDMICF_3SO_3$ (◀) and TMACl (★) showing in **a**: the electrical Energy U_e, in **b**: the potential E_{red} at reduction and in **c**: the linear strain ε against the current density j at reduction.

© 2020 by the authors. Submitted for possible open access publication under the terms and conditions of the Creative Commons Attribution (CC BY) license (http://creativecommons.org/licenses/by/4.0/).
